# Short-Term Effects of Tourmaline on Nitrogen Removals and Microbial Communities in a Sequencing Batch Reactor at Low Temperatures

**DOI:** 10.3390/ijerph15061280

**Published:** 2018-06-17

**Authors:** Yahong Han, Shan Qiu, Hongyun Zeng, Fang Ma, Jue Wang, Yilun Qiu, Xuedi An

**Affiliations:** 1 School of Environment Harbin Institute of Technology, No. 73, the Yellow River Road, Nangang District, Harbin 150090, China; hyahonghong@126.com (Y.H.); mafang@hit.edu.cn (F.M.); wangjue299@hit.edu.cn (J.W.); LL2012323@126.com (Y.Q.); 16S127181@stu.hit.edu.cn (X.A.); 2 State Laboratory of Urban Water Resources and Environment Harbin Institute of Technology, No. 73, the Yellow River Road, Nangang District, Harbin 150090, China; 3 Heilongjiang Provincial Environmental Science Research Institute, Harbin 150056, China; zenghongyun_hky@163.com (H.Z.)

**Keywords:** low temperatures, microbial community, nitrogen removal, tourmaline

## Abstract

Tourmaline is a ring borosilicate with unique pyro-electricity and piezoelectricity values. Non-gem tourmaline is usually used as an environmental material. The short-term effects of ultrafine tourmaline particles on nitrogen removal performs microbial population dynamics. Key functional species in a sequencing batch reactor were investigated at 9 ± 1 °C. The investigation results showed that 1 g·L^−1^ ultrafine tourmaline particles could resist the effect of temperature shock on the metabolism of NH_4_^+^-N and were beneficial to the restoration of the metabolism capacity of NH_4_^+^-N. 1 g·L^−1^ ultrafine tourmaline particles, which increased the oxidation rate of NH_4_^+^-N in the aerobic phase, the formation rate of NO_3_^−^-N in the aerobic phase, and the denitrification rate in the hypoxia phase at low temperatures. However, the community richness or diversities were not changed after short-term exposure to 1 g·L^−1^ ultrafine tourmaline particles at low temperatures and 1 g·L^−1^ ultrafine tourmaline particles could not change the relative abundances of functional microbes except nitrite oxidizing bacteria.

## 1. Introduction

Tourmaline is a ring borosilicate with unique pyro-electricity and piezoelectricity values [1]. The composition and origin of tourmaline determine its crystalline shape and color. Tourmaline with a perfect crystalline shape is called gem tourmaline. Non-gem tourmaline have a poor crystalline shape or blocky texture, which is usually used as environmental materials. The most important feature of tourmaline is the spontaneous and permanent poles, which provide an electric dipole especially in a small granule with several micrometers or smaller [2]. Therefore, there is a strong electric field on the surface of a tourmaline granule [3]. The electric field showed positive effects on the growth and metabolism of organisms [4]. The growth of sulfate reducing bacteria was promoted by nanometer/submicron tourmaline [5]. Tourmaline could promote the growth of Caco-2 cells and the activity of alkaline phosphatase [6]. The low concentration of tourmaline could stimulate the proliferation of *E. coli* [7]. Recent studies suggested that tourmaline promoted the growth and metabolism of anammox bacteria [8] and increased the removal rates of ammonia nitrogen (NH_4_^+^-N) and total nitrogen (TN) in reactors [9]. However, the effect of tourmaline on the activated sludge after short-term exposure to ultrafine tourmaline particles (UTPs) at low temperatures is still unknown.

In Northeastern China, the wastewater temperature became very low during the winter. Therefore, the effluent concentrations of nitrogen in wastewater treatment plants increase, which leads to the problems of eutrophication and threats to human health [10]. In biological wastewater treatment processes, low temperatures may cause several problems such as higher sludge production, more filamentous microorganisms, lower sludge settle ability, and worsening effluent quality [11]. In addition, temperature is an important parameter for determining the microbial metabolism and community structure in all environments [12]. In traditional processes, nitrogenous compounds are removed from wastewater through the combination of sequential nitrification and denitrification processes [13] and the biotransformation processes are decreased largely in nitrification and denitrification at low temperatures [14]. For instance, the removal rate of ammonia nitrogen (NH_4_^+^-N) was about 90% at 15 °C in the activated sludge treatment system and it decreased to about 20% at 10 °C and to 0% at 5 °C [15]. Additionally, the effluent quality turned worse in wastewater treatment for excessive growth of filamentous microorganisms at 8 °C [16]. Therefore, it is necessary to reduce the effects of temperatures on wastewater treatment systems.

Previous publications reported that tourmalines had positive effects on the reaction rates at room temperature [17]. Therefore, we attempted to investigate the effects of tourmalines at low temperatures in order to reduce the effects of low temperatures on wastewater treatments. The study aimed to assess short-term effects of ultrafine tourmaline particles (UTPs) on activated sludge in sequencing batch reactors (SBRs) including biological nitrogen removal and bacterial population dynamics at low temperatures in wastewater treatment plants. Moreover, Illumina MiSeq techniques were used to study the effects of UTPs on the microbial community structure at low temperatures in order to find the key factors for the removal of nitrogen.

## 2. Materials and Methods

### 2.1. Ultrafine Tourmaline Particles

The UTPs used in this study were purchased from Tianjin Hongyan Tianshan Mining Nano-Tech CO., Ltd. and the average size of UTPs was 4.382 ± 0.004 μm. The UTPs used in the experiment belong to magnesium tourmaline, which was confirmed by Transform Infrared Spectroscopy (PerkinElmer, Norwalk, CT, USA) and the field emission scanning electron microscopy (ZEISS, Jena, Germany).

### 2.2. SBRs with Short-Term Exposure to UTPs at Low Temperatures

The parent SBR was operated over 100 days at room temperatures with the effluent concentrations as follows: 6.10 ± 0.7 mg·L^−1^ NO_3_^−^-N, 0.09 ± 0.02 mg·L^−1^ NO_2_^−^-N and 0.19 ± 0.03 mg·L^−1^ NH_4_^+^-N. The working volume was 5 L. The concentration of dissolved oxygen was 2.2 ± 0.2 mg·L^−1^. The concentration of mixed liquor volatile suspended solids (MLVSS) was about 3000 ± 130 mg·L^−1^. Solid retention time was 20 d. The cycle time was 8 h, which included influent period (15 min), anaerobic period (1 h), aerobic period (2.5 h), hypoxia period (2.5 h), settling period (0.5 h), decanting period (15 min), and idle period (1 h). The activated sludge of offspring SBRs came from the parent SBR and the operating conditions of offspring SBRs were the same as parent SBR except for the temperature. The offspring SBRs were divided into two groups, which includes the test group with 1 g·L^−1^ UTPs and the control group without 1 g·L^−1^ UTPs. Each group had three parallel SBRs. The operating temperature of the two groups was decreased suddenly from room temperature to 9 ± 1 °C in a self-designed low-temperature laboratory and short-term phase referred to as 7-day operation in the above conditions. After the sludge discharged, UTPs was added according to the discharge ratio of the sludge. The effluents were sampled every three cycles in 7 days and the samples were collected at 5 cm under the liquid level every 30 min at the first cycle of the eighth day. The effluents and the mixtures without activated sludge were tested with the concentrations of NH_4_^+^-N, NO_3_-N, and NO_2_^−^-N. Synthetic wastewater was used in this study and the initial concentrations of chemical oxygen demand (COD), NH_4_^+^-N, NO_2_^−^-N, and NO_3_^−^-N were 600 ± 12.4 mg·L^−1^, 55 ± 2.8 mg·L^−1^, 0.5 ± 0.12 mg·L^−1^ and 4.8 ± 0.34 mg·L^−1^ at 9 ± 1 °C in the synthetic wastewater, respectively. The initial pH of synthetic wastewater was 7.5 and it was not kept constant.

### 2.3. DNA Extraction and PCR

At low temperatures, the activated sludge of short-term operation was at a lag phase. Therefore, we considered that the microbial community was constant in the structure and the biomass was in the anaerobic phase, aerobic phase, and hypoxia phase. DNA was extracted from activated sludge of SBRs with the kit of PowersoilTM (MOBIO, San Diego, CA, USA) and the activated sludge was obtained at the end of the idle period. It was washed 3 times with deionized water by centrifugation at 4000 g for 5 min in both groups after short-term operation. The PCR primers used to amplify 16S rRNA genes were 515F (5′-AGAGTTTGATCCTGGCTCAG-3′) and 907R (5′-CCGTCAATTCMTTTRAGTTT-3′) with a unique six-base barcode inserted at the 5′ end of the forward primer to distinguish each sample [18]. The PCR reaction mixture consists of 2 µL of 2.5 mmol·L^−1^ dNTPs, 4 µL of 5X FastPfu Buffer, 0.8 µL of each primer (5µmol·L^−1^), 0.4 µL of Fastpfu Polymerase, 10 ng of template DNA, and sterile double-distilled. The PCR reaction conditions were programmed as follows: initial denaturation at 95 °C for 3 min, then 30 cycles of denaturation at 95 °C for 30 s, annealing at 55 °C for 30 s, extension at 72 °C for 1 min, and final extension at 72 °C for 5 min.

### 2.4. Illumina MiSeq

Amplification products were loaded on an Illumina MiSeq platform by a commercial company (Majorbio, Shanghai, China), according to the protocols. The raw data of sequences were assembled with Trimmomatic software (Version 0.32, Aachen, Germany). The high-quality sequences were classified into operational taxonomic units (OTUs) with UPARSE software (Version 7.1, Tiburon, CA, USA), according to a sequence similarity threshold of 97% [19]. The Chao1 estimator, ACE estimator, Shannon index, Simpson index, and Good’s coverage were analyzed with Mothur software (Version 1.30.1, Ann Arbor, MI, USA). The R language platform of the VEGAN package (Auckland, New Zealand) was used to discover the microbial diversities and abundance datasets from each group. The raw data were deposited in the Sequence Read Archive Database of NCBI (National Center for Biotechnology Information) with the accession number SRP111300.

### 2.5. Scanning Electron Microscopy (SEM)

According to the preparation method of samples for SEM, the samples were taken after 7 days and 5 mL of sludge was first washed 3 times with 0.1 mol·L^−1^ phosphate buffer (pH 7.4) by centrifugation at 12,000 g for 5 min. Then the sludge was fixed in 2.5% glutaraldehyde for 4 h at 4 °C. After washing three times with 0.1 mol·L^−1^ phosphate buffer (pH 7.4), the sludge was dehydrated in a graded ethanol series (50%, 70%, 80%, 90%, and 100%, 15 min in each step) and dried in the air. The obtained samples were observed by the field emission scanning electron microscopy (ZEISS, GER).

### 2.6. Analytical Methods

The cell proliferation assays of UTPs to activated sludge were measured with the cell counting kit-8 (Dojindo, Kumamoto-ken, Japan), according to the literature [20]. In the experiment, the test group included the re-suspended sludge, the CCK-8 solution, and 1 g·L^−1^ UTPs in each well of 96-well plates. The controls had two groups (denoted C1 and C2), C1 was composed of the re-suspended sludge and CCK-8 solution while C2 consisted of the re-suspended sludge, CCK-8 solution, and 1 g·L^−1^ glass beads with average particle size of 4.5 ± 0.2 µm. The concentrations of NH_4_^+^-N, NO_3_-N, and NO_2_^−^-N were measured by ultraviolet spectrophotometry [21]. Each test was performed in triplicate in this study. The significance of the results was verified by an analysis of variance (ANOVA) and *p* < 0.05 was considered statistically significant. Removal efficiencies (RE) as well as removal rates (RR) were calculated using the equations below.

(1) $$\mathrm{RE}=\frac{C_{0}-C_{t}}{C_{x}}\times 100\%,$$

(2) $$\mathrm{RR}=\frac{C_{0}-C_{t}}{C_{x}\cdot t},$$

where *C*_0_ and *C*_t_ are respectively the initial concentration and the concentration at time t and the unit is mg·L^−1^. *t* is the treatment time of the activated sludge and the unit is h. *C*_x_ is the concentration of MLVSS and the unit is mg·(g·h)^−1^ [22].

## 3. Results and Discussion

### 3.1. Effects of UTPs on Effluent at Low Temperatures

**Figure 1:**
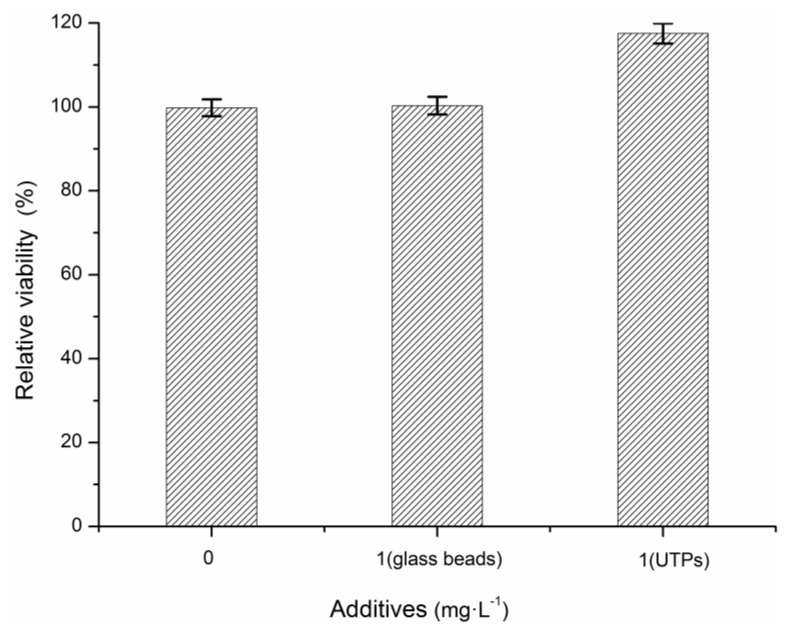
Relative viability of activated sludge with the presences of 0 g·L^−1^ UTPs, 1 g·L^−1^ glass beads, and 1 g·L^−1^ UTPs.

Cell proliferation assays were adopted to investigate the possible effects of UTPs on sludge viability [23]. As shown in Figure 1, the viability of the activated sludge was increased significantly in the presence of 1 g·L^−1^ UTPs. However, there were no significant differences in the relative viabilities of activated sludge between C1 and C2 (*p* > 0.05), which indicates that the effects of 1 g·L^−1^ UTPs on activated sludge were irrelevant to the appearance of morphological characteristics of UTPs.

**Figure 2:**
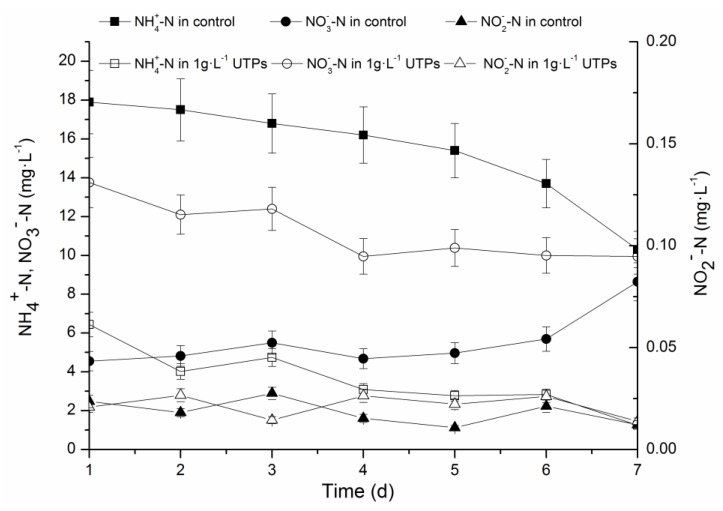
Concentrations of NH_4_^+^-N, NO_2_^−^-N, and NO_3_^−^-N in effluents over short-term operation. (The influent concentrations of NH_4_^+^-N, NO_2_^−^-N, and NO_3_^−^-N were 55 ± 2.8 mg·L^−1^, 0.5 ± 0.12 mg·L^−1^ and 4.8 ± 0.34 mg·L^−1^ at 9 ± 1 °C, respectively).

The effect of short-term exposure to 1 g·L^−1^ UTPs on biological nitrogen removal at low temperatures is not well understood yet. The effluent concentrations of NH_4_^+^-N were gradually decreased in both groups (Figure 2), which decreased from 6.44 ± 0.32 mg·L^−1^ to 1.25 ± 0.08 mg·L^−1^ in the test group and from 17.93 ± 0.87 mg·L^−1^ to 10.30 ± 0.61 mg·L^−1^ in the control group. However, the effluent concentrations of NH_4_^+^-N in both groups were still statistically higher (*p* < 0.05) than in the parent SBR (0.19 ± 0.03 mg·L^−1^) during the seventh day. The effluent concentration of NH_4_^+^-N in the test group was much lower than the one in the control group. Compared with the first cycle respectively, the effluent concentration of NH_4_^+^-N was decreased by 80.62 ± 4.2% in the test group and 42.51 ± 2.55% in the control group when compared to the last cycle at the seventh day. It was illustrated that 1 g·L^−1^ UTPs were conducive to resisting the effect of temperature shock on metabolism of NH_4_^+^-N. Moreover, 1 g·L^−1^ UTPs were beneficial to the restoration of the metabolism capacity of NH_4_^+^-N in short-term exposure at 9 ± 1 °C. With the decrease in the effluent concentration of NH_4_^+^-N, the effluent concentration of NO_3_^−^-N was decreased in the test group but increased in the control group. The effluent concentration of NO_3_^−^-N was decreased from 13.75 ± 0.72 mg·L^−1^ to 9.95 ± 0.46 mg·L^−1^ in the test group and increased from 4.55 ± 0.23 mg·L^−1^ to 8.64 ± 0.44 mg·L^−1^ in the control group during the 7-day operation. Although the effluent concentrations of NH_4_^+^-N and NO_3_^−^-N were changed significantly, the effluent concentrations of NO_2_^−^-N in both groups were lower than 0.027 mg·L^−1^ at all times, which indicates that 1 g·L^−1^ UTPs had no measurable effect on the effluent concentration of NO_2_^−^-N after 7-day operation at 9 ± 1 °C. The change trends of nitrogen showed that 1 g·L^−1^ UTPs could accelerate the denitrification process during short-term exposure at low temperatures.

**Figure 3:**
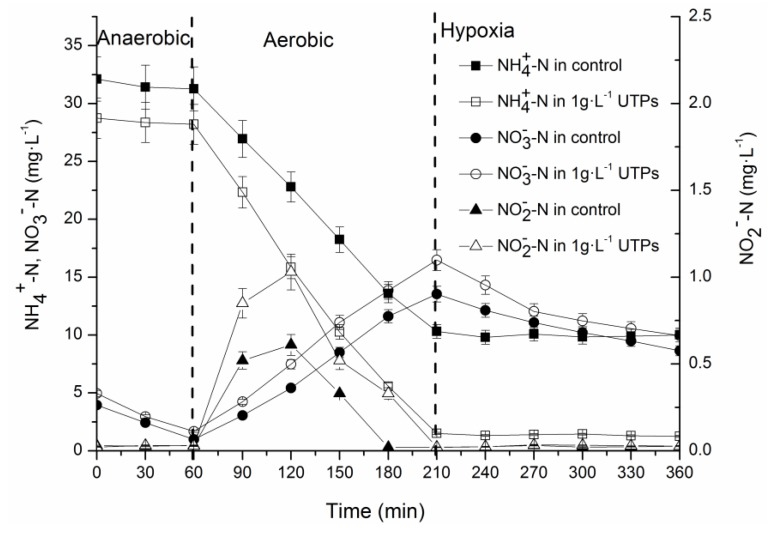
Variations of the concentrations of NH_4_^+^-N, NO_2_^−^-N, and NO_3_^−^-N within one cycle after short-term operation.

In this study, concentrations of nitrogen in three phases including the anaerobic phase, aerobic phase, and hypoxia phase were used to investigate short-term effects of 1 g·L^−1^ UTPs at 9 ± 1 °C in the last cycle of 7-day operation. In the anaerobic phase, the initial concentrations of NH_4_^+^-N and NO_3_^−^-N of the control group were different from those in the test group (Figure 3), which was caused by the undischarged sewage of the last cycle. The concentrations of NO_3_^−^-N in both groups were low for denitrification in the anaerobic phase. In the aerobic phase, the oxidation rate of NH_4_^+^-N was 2.79 ± 0.2 mg·(g·h)^−1^ in the control group and 3.56 ± 0.26 mg·(g·h)^−1^ in the test group. The formation rate of NO_3_^−^-N was 1.67 ± 0.12 mg·(g·h)^−1^ in the control group and 1.97 ± 0.29 mg·(g·h)^−1^ in the test group. The accumulation rates of NO_2_^−^-N were low in two groups, which indicates that the effect of low temperatures on ammonia oxidizing bacteria (AOB) was greater than that on nitrite oxidizing bacteria (NOB). In the hypoxia phase, the denitrification rate was 0.65 ± 0.06 mg·(g·h)^−1^ in the control group and 0.87 ± 0.08 mg·(g·h)^−1^ in the test group. The differences illustrated that 1 g·L^−1^ UTPs increased the oxidation rate of NH_4_^+^-N in the aerobic phase, the formation rate of NO_3_^−^-N in the aerobic phase, and the denitrification rate in the hypoxia phase at 9 ± 1 °C. Since the free ammonia can inhibit the nitrification and the inhibitory effect was increased with the increase of the concentration of free ammonia [24], the higher concentration of NH_4_^+^-N in the control group further slowed down the nitrification rate when compared to the test group. Taking into account the constraints of the initial NH_4_^+^-N concentrations and reaction time, despite the very similar curves, their difference was significant since it was seen in all three batches that the reaction rates of the first half of the aerobic phase were significantly larger than that of the second half with the consumption of reaction substrates. In addition, high concentration of NH_4_^+^-N remained in the control group at the end of the aerobic phase. As a matter of fact, it did not reachthe steady state after 7 days of operation. Therefore, the changes observed indicated that tourmaline contributed to the adaptation of activated sludge to low temperatures. It was reported that tourmaline particles with a diameter less than 1 μm can provide electron donors for denitrification [25]. Therefore, the denitrification rate in the test group was higher than that in the control group, which suggests that 4.382 ± 0.004 μm tourmaline might also provide electrons for denitrification.

**Figure 4:**
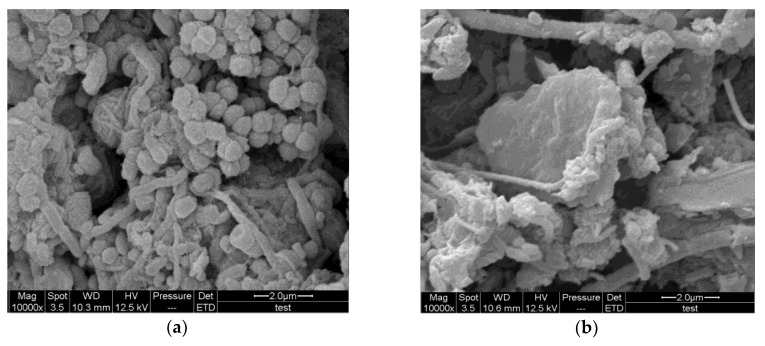
(**a**) SEM of the activated sludge in the control group. (**b**) SEM of the activated sludge in the test group.

In this study, SEM analysis was performed to assess the surface structure of activated sludge. A large number of cocci-shaped cells and filamentous bacteria were observed in all activated sludge samples (Figure 4). The sludge structure with strong bonding between bacteria (Figure 4a) was compact while the sludge structure of highly porous biofilm (Figure 4b) was loose. However, there were no effect on the surfaces of cocci-shaped cells and filamentous bacteria in both groups by 1 g·L^−1^ UTPs at low temperatures. In the settling period of SBRs, UTPs were precipitated and mixed with sludge, but microorganisms were not attached to UTPs (Figure 4b). This may be because a strong electric field intensity related to the distances on the surface of a tourmaline granule [3] prevented the attachment. However, previous studies showed that the stimulation of the electric field could enrich microorganisms during the treatment [26] and tourmaline particles with a diameter less than 1 μm adhering to the surface of bacteria during the treatment [25]. Therefore, the effect of 1 g·L^−1^ UTPs on microorganisms might be related to the size of tourmaline and the distances between microorganisms and tourmaline.

### 3.2. Bacterial Community Shift in Activated Sludge after Short-Term Exposure to UTPs at Low Temperatures

**Figure 5:**
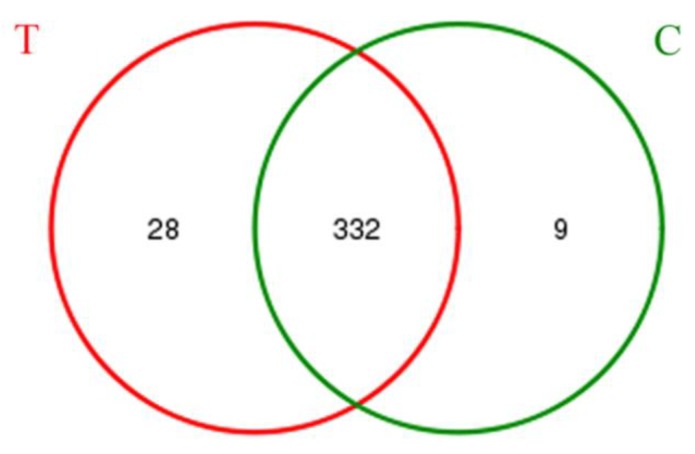
OUT VENN analysis. T stands for the test group and C stands for the control group.

**Figure 6:**
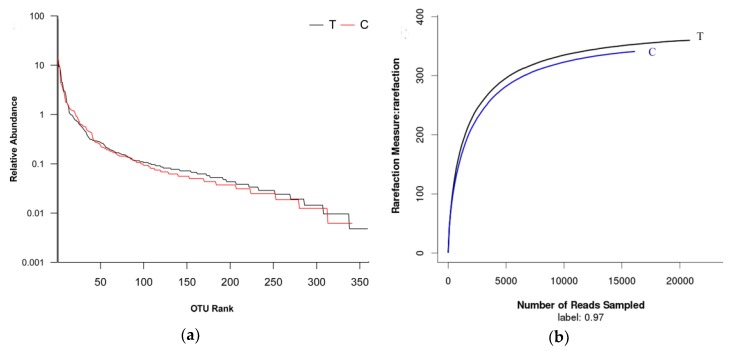
(**a**) Rarefaction curves. (**b**) OTUs Rank-Abundance curves. T stands for the test group and C stands for the control group.

The technology of MiSeq pyrosequencing was used to analyze the microbial diversity and community structure in two groups. A total of 47,146 raw sequences were obtained after pyrosequencing. After processing these raw sequences, the recovered quantity of 16S rRNA was 36,931 and the average length was about 445 bp. These sequences were classified into 360 OTUs in the test group and 341 OTUs in the control group, respectively. Apparently, the test group and control group shared 89.97% of the total OTUs (Figure 5). The coverage estimators of two groups were all above 0.998 (Table 1), which illustrates that the obtained sequences could cover the microbial diversity of activated sludge systems [27]. Both bacterial community richness and community diversity shared the high similarities between the test group and control group (Table 1), which indicates that 1 g·L^−1^ UTPs had no impact on bacterial community richness and diversity in activated sludge after short-term operation at 9 ± 1 °C. The above results were also supported by rank-abundance curves and rarefaction curves (Figure 6).

**Figure 7:**
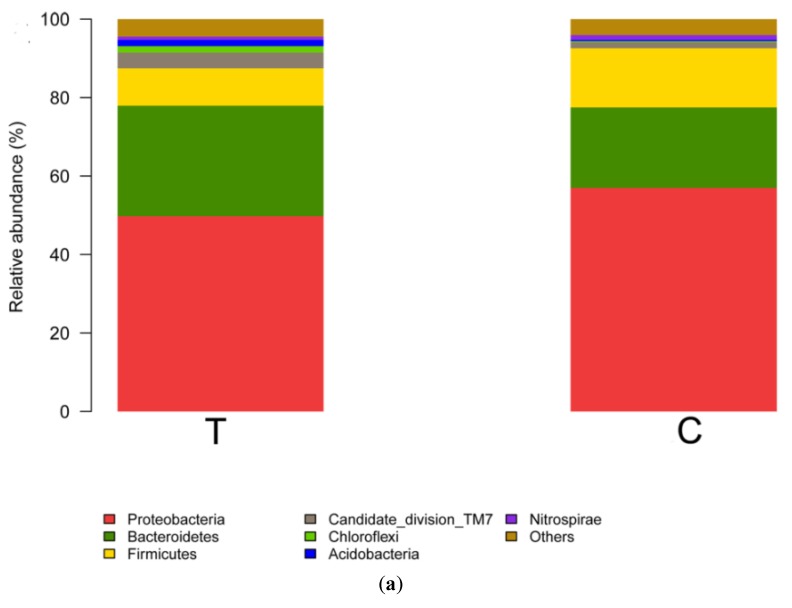

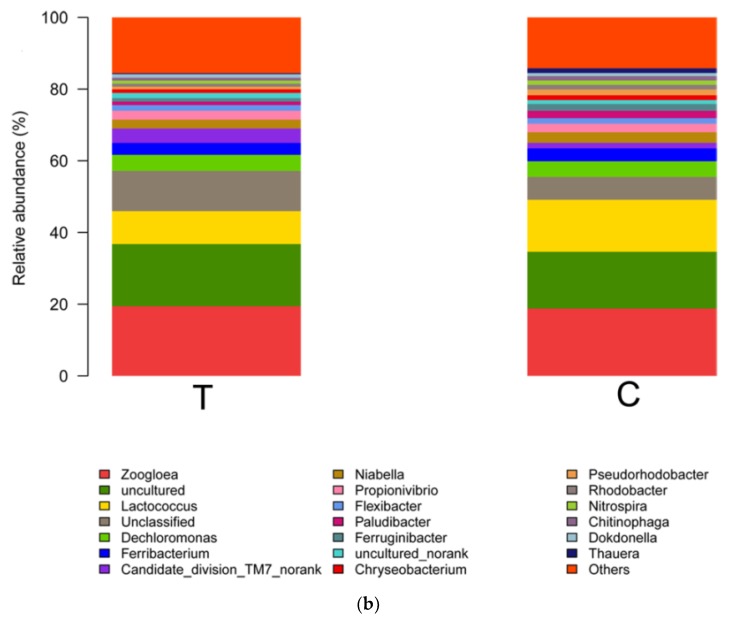
(**a**) Microbial community barplot at the levels of phylum after short-term operation. (**b**) The microbial community bar plot at the levels of genus after short-term operation. T stands for the test group and C stands for the control group.

The relative abundances of the activated sludge at phylum and genus levels were shown in Figure 7 and the activated sludge were collected at the last cycle during short-term operation. The top two predominant phyla were the same in the test group and control group, which were Proteobacteria and Bacteroidetes and, respectively, accounted for 49.76% and 28.19% in the test group and 56.98% and 20.52% in the control group (Figure 6a). The other phyla contained Firmicutes (9.51%), Candidate (4.21%), Chloroflexi (1.64%), Acidobacteria (1.59%), Verrucomicrobia (0.93%), Nitrospirae (0.83%), and others (3.35%) in the test group and Firmicutes (15.08%), Candidate (2.42%), Nitrospirae (1.22%), BD1-5 (0.74%), Chlorobi (0.73%), and others (2.32%) in the control group. The maximum difference in the relative abundance between two groups was 7.67% derived from Bacteroidetes, but the shift of the microbial community structure was not significant (*p* > 0.05) at the phylum level. The analysis at the level of the genus was operated based on the top 24 abundant genera (Figure 6b). The most predominant genus was *Zoogloea* (19.41%) in the test group and *Zoogloea* (18.77%) in the control group. Although most of the major genera had some changes in relative abundance among microbial communities, the microbial community structure did not change significantly (*p* > 0.05) after short-term operation. Overall, the detected microorganisms were typical microbes in activated sludge in SBRs, according to previous research studies [28]. It was concluded that the effect of 1 g·L^−1^ UTPs on the nitrogen removal rate was not achieved by changing the microbial community structure. The electrical stimulation produced by the permanent electrodes of tourmaline may be the true reason for the changing nitrogen removal rate.

### 3.3. Key Functional Species

The nitrogen removal process in wastewater involves nitrification and denitrification and the key functional microbes in nitrogen removal include AOB, NOB, and denitrifying bacteria (DNB). The composition of the functional microbial community related to nitrogen removal was shown in Table 2. The shift of key functional microbes in relative abundances was used to explain the effect of tourmaline on nitrogen removal after short-term operation. The only AOB was *Nitrosomonas* and the relative abundance of *Nitrosomonas* was 0.19% in both groups. The concentrations of NH_4_^+^-N in effluent in both groups were higher than that in the parent SBR (0.19 mg·L^−1^) after short-term operation, which indicated that low temperatures reduced the metabolism rate of AOB to NH_4_^+^-N. The concentration of NH_4_^+^-N in effluent in the control group was higher than that in the test group, which suggested that 1 g·L^−1^ UTPs reduced the sensitivity of AOB to low temperatures. *Nitrospira* was the unique NOB in the two groups and the relative abundances of *Nitrospira* decreased from 1.22% in the control group to 0.83% in the test group, which illustrated that 1 g·L^−1^ UTPs inhibited the proliferation of NOB after short-term operation. It is reported that the optimum pH for the growth of NOB was 7.7 to 7.9 [29]. Therefore, the pH (7.7) in the control group was conducive to the proliferation of NOB and the pH (8.2) in the test group was slightly farther from the optimum pH of NOB. This explained why the relative abundance of NOB in the test group was smaller than that in the control group after short-term exposure to 1 g·L^−1^ UTPs at 9 ± 1 °C.

Relative abundance of DNB had the minimal change from 22.77% in the control group to 21.56% in the test group after short-term operation, which indicated that 1 g·L^−1^ UTPs had no effect on the relative abundance of DNB at low temperatures. Among DNB, *Zoogloea*, which is a common bacteria in sludge samples [30], was an absolute predominant genus and accounted for 90.11% of the DNB in the test group and 82.45% of the DNB in the control group, respectively. After 7 days operation, MLVSS in the control group was 2920 ± 90 mg·L^−1^ while MLVSS in the test group was 3060 ± 100 mg·L^−1^. The difference in the biomass of the two groups was not significant since the two groups were both in the lag phase. In short, low temperatures reduced the metabolism rate of functional microbes related to nitrogen removal and 1 g·L^−1^ UTPs could not change the relative abundances and biomass of functional microbes significantly except NOB after short-term exposure to 1 g·L^−1^ UTPs at 9 ± 1 °C. In addition, the generation period of NOB was shorter than that of AOB at low temperatures [31], which was also a reason for the significant difference in NOB.

## 4. Conclusions

This study demonstrated that 1g·L^−1^ UTPs resist the effect of temperature shock on the metabolism of NH_4_^+^-N and were beneficial for restoring the metabolism capacity of NH_4_^+^-N. In summary, 1 g·L^−1^ UTPs increased the oxidation rate of NH_4_^+^-N and the formation rate of NO_3_^−^-N in the aerobic phase and accelerated the denitrification rate in the hypoxia phase after short-term exposure to 1 g·L^−1^ UTPs at low temperatures. The community richness or diversities were not changed by 1 g·L^−1^ UTPs and 1 g·L^−1^ UTPs could not change the relative abundances of functional microbes significantly except NOB after short-term exposure at low temperatures. Tourmaline can be applied to start up the reactors quickly and recover the reactor operation quickly at low temperatures.

## Figures and Tables

**Table 1:** Similarity-based OTUs and bacterial community diversity indices based on Illumina MiSeq sequencing bacterial data (at the sequence similarity of 97%).

Samples ID	Reads	OTU	ACE	Chao	Coverage	Shannon	Simpson
The test group	20,839	360	369 (364, 381)	368 (363, 383)	0.998896	4.07 (4.05, 4.09)	0.0433 (0.0422, 0.0444)
The control group	16,092	341	353 (346, 366)	353 (346, 371)	0.998198	3.97 (3.94, 4)	0.0501 (0.0485, 0.0517)

**Table 2:** Relative abundances of functional species at the genus level.

Functions	Taxa	The Test Group	The Control Group
AOB	*Nitrosomonas*	0.19%	0.19%
NOB	*Nitrospira*	0.83%	1.22%
DNB	*Zoogloea*	19.41%	18.77%
	*Thauera*	0.26%	1.30%
	*Rhodobacter*	0.85%	1.26%
	*Arcobacter*	0.19%	0.61%
	*Acinetobacter*	0.56%	0.34%
	*Flavobacterium*	0.20%	0.42%
	*Comamonas*	0.03%	0.06%
	*Hyphomicrobium*	0.04%	0.02%
